# Supplementary material for: Running style and stability during uphill running are largely preserved with increasing shoe sole thickness
Source: PLoS One. 2026 Oct 8;21(10):e0348380. doi: 10.1371/journal.pone.0348380 (PMC13649003; doi:10.1371/journal.pone.0348380)
Supplement: Supplementary file 2 [file pone.0348380.s007.docx]

*Editor:* ***Comments to the Author***

*The manuscript addresses an interesting topic, but the reviewers raised important concerns regarding the study rationale, participant selection, footwear-condition description, testing protocol, and interpretation of the stability results. Please revise the manuscript carefully and provide a clear point-by-point response to all reviewer comments. Thanks.*

**Our response:**
The authors thank the editor and reviewers for the critical and thorough revision of our work. We revised our manuscript carefully to address the criticized points. We hope that we successfully addressed all the aspects. We highlighted the new changes we made addressing all comments.

*Reviewer #1:* ***Comments to the Author***

***Reviewer’s response:***

**Our response:**
The authors thank the editor and reviewers for the critical and thorough revision of our work. We revised our manuscript carefully to address the criticized points. We hope that we successfully addressed all the aspects. We highlighted the new changes we made addressing the Reviewer #1’s comments with yellow. Please see below for our responses to each point. Please note that the line numbers are abbreviated with L and refer to the revised manuscript with tracked changes.

***Reviewer’s response:***

*Line 100: The purpose of your study appears to be for the implication of shoe design and injury prevention; however, you do not specify what kind of injury. In the introduction ankle injuries are mentioned, but there is no research cited that provides context and or relevance to the frequency or likelihood of ankle injuries occurring in uphill running. Please revise to specify the type of injury prevention. If ankle injury is the primary injury, include justification to the relevance ankle injuries occurring in uphill running (e.g. frequency or mechanism).*

**Our response:**
Thank you very much for this comment. We agree that the original wording was too broad, as the present study did not directly assess injury occurrence or injury prevention. We therefore revised the Introduction to soften this implication and to clarify that the injury-related relevance of the study concerns potential ankle-stability-related injury risk rather than injury prevention in a direct clinical sense. Because direct epidemiological evidence linking uphill running specifically to ankle injury occurrence is limited, we avoided overstating this point. Instead, we added context that ankle injuries are relevant in trail and mountain running populations, where uphill running is a common task demand, and explained the biomechanical relevance of ankle stability in relation to increased sole thickness.

A systematic review reported the ankle as one of the common injury sites in trail runners and ligament sprains among common injury diagnoses (Viljoen et al., 2021), and a study in young trail runners reported the ankle as the most frequently affected injury region (Sanchez-Garcia et al., 2022).

**References for our response:**

Sanchez-Garcia, L. F., Penichet-Tomas, A., Pueo, B., & Jimenez-Olmedo, J. M. (2022). Injury Incidence and Pattern in Elite Young Male and Female Trail Runners. Applied Sciences (Switzerland), 12(3). <https://doi.org/10.3390/app12031155>

Viljoen, C. T., Janse van Rensburg, D. C., Verhagen, E., van Mechelen, W., Tomás, R., Schoeman, M., Scheepers, S., & Korkie, E. (2021). Epidemiology of Injury and Illness Among Trail Runners: A Systematic Review. Sports Medicine, 51(5), 917–943. https://doi.org/10.1007/s40279-020-01418-1

**Changes in manuscript:**

We revised the relevant paragraph of the Introduction as follows: “Despite the practical importance of uphill running in disciplines such as trail running and mountain racing, the isolated effects of shoe sole thickness under uphill conditions have not yet been systematically examined. Previous studies investigating shoe effects during uphill running have typically compared shoe models differing in multiple design characteristics, making it difficult to attribute observed effects specifically to sole thickness [38–40]. A better understanding of how sole thickness influences running mechanics during uphill locomotion may therefore provide valuable insights for performance-oriented shoe design and for interpreting ankle-stability-related biomechanical responses. Although the present study did not assess injury occurrence, this ankle-specific perspective is relevant because ankle injuries and ligament sprains are commonly reported in trail-running populations, where uphill running, downhill running, and uneven terrain are frequent task demands [41,42]. From a biomechanical perspective, increased sole thickness may affect frontal-plane ankle mechanics and lateral stability by increasing the distance between the foot and the ground [9,12,13].” (L 94-106)

***Reviewer’s response:***

*Line 114: You’ve stated that only male participants were included in the study due to the limited shoe size of US men’s 9. This is not a sound justification. A US men’s 9 is the equivalent of a US women’s 10.5 shoe size which would allow some female participants to be included. Please provide a better justification.*

**Our response:**
Thank you for this comment. We agree that the original wording was imprecise. We did not intend to imply that a US men’s 9 shoe size categorically excludes all female participants. Rather, the standardized test shoes were available only in one size, and recruitment was restricted a priori to male runners who fit the available standardized shoes in order to ensure consistent shoe fit across conditions and to reduce heterogeneity related to sex-specific running biomechanics (Xie et al., 2022) and foot morphology (Krauss et al., 2008). We have revised the Methods section to clarify this point. We also acknowledge that the inclusion of only male runners limits the generalizability of the findings, and this limitation has been stated in the Limitations section.

**References for our response:**

Krauss, I., Grau, S., Mauch, M., Maiwald, C., & Horstmann, T. (2008). Sex-related differences in foot shape. Ergonomics, 51(11), 1693–1709. <https://doi.org/10.1080/00140130802376026>

Xie, P. P., István, B., & Liang, M. (2022). Sex-specific differences in biomechanics among runners: A systematic review with meta-analysis. Frontiers in Physiology, 13. <https://doi.org/10.3389/fphys.2022.994076>

**Changes in manuscript:**
We revised the relevant section as follows: “The standardized test shoes were available only in one size (US men’s 9). To ensure consistent shoe fit across conditions and reduce heterogeneity related to sex-specific running biomechanics [43] and foot morphology [44], recruitment was restricted a priori to male runners who fit the available standardized shoes.” (L 122-6)

***Reviewer’s response:***

*Lines 121-131: Information about the foam composition between the AFTs and the control shoes is not provided. Are the foam compositions the same? If so, please state this in your methods and introduction as differing compositions would influence comparisons between control and AFT. Further if the foam compositions are different between the control shoe and the AFT shoes there would be no need for the control shoe. What is the justification for comparing the two AFT conditions to a control shoe? If the goal of the study was to isolate the effect of sole thickness, and the other elements between AFT shoes remain the same, you can make comparisons between the two AFT conditions directly.*

**Our response:**
Thank you for this important comment. We agree that the original description of the shoe conditions did not sufficiently clarify the foam composition and the rationale for including the control shoe. The two AFT shoes had the same advanced midsole foam composition and carbon-based propulsion elements, whereas the conventional control shoe had a traditional midsole construction. CON27 was included as a conventional reference condition to allow comparison between AFT shoes and a traditional lower-stack running shoe. In contrast, the direct comparison between AFT35 and AFT50 was used to examine thickness-related effects within the AFT design, because these two shoes were identical in construction apart from sole thickness. We have revised the Methods section accordingly.

**Changes in manuscript:**

The relevant part in the Methods section is revised to: “The two AFT models incorporated the same advanced midsole foam composition and carbon-based propulsion elements, whereas CON27 represented a conventional lower-stack running shoe with a traditional midsole construction [14]. To minimize confounding effects, shoes were matched as closely as possible in mass (< 50 g difference) and heel-to-toe drop (7–8 mm). CON27 was included as a conventional reference condition to allow comparison between AFT shoes and a traditional lower-stack running shoe. Because AFT35 and AFT50 were identical in construction apart from sole thickness, the direct comparison between these two models was used to examine thickness-related effects within the AFT design.” (L 138-46)

***Reviewer’s response:***

*Line 139: The familiarization phase on a treadmill takes 3 minutes and this is cited in your methods. Why did you include a 2-minute walking phase after the 3 minutes of familiarization were already reached? Please provide a justification for this.*

**Our response:**
Thank you for this comment. We agree that the purpose of the additional 2-min walking phase was not sufficiently clear. The 3-min running period represented the actual shoe-specific treadmill running familiarization, following the cited recommendation. The subsequent 2-min walking phase was not intended to extend the running familiarization period, but served as a standardized low-intensity transition and active recovery phase between shoe blocks. This allowed participants to remain accustomed to the respective shoe condition while minimizing fatigue before the experimental trials. We have revised the Methods section to clarify this point.

**Changes in the manuscript:**

We revised the relevant section as follows: “Each block began with a 5-min shoe-specific familiarization [48], consisting of 3 min running and 2 min walking at self-selected speed under level condition. The walking phase served as a standardized low-intensity transition before the experimental” (L 158-61)

***Reviewer’s response:***

*Line 145: You’ve referenced 3 prior studies of your own that reported the results of downhill and level running, and in the current manuscript you are reporting the results for uphill running. Why not report all findings in the same paper? If it was the same methods, protocol, and participants it seems redundant to report the findings in three separate studies. This is also a form of self-plagiarism that can lead to manuscript rejection without proper justification.*

**Our response:**
Thank you for raising this important point. We understand the concern regarding redundancy and potential overlap between manuscripts. The level, downhill, and uphill conditions were collected within the same broader experimental project. However, the present study addresses a distinct task-specific research question focused on uphill running. Uphill, level, and downhill running differ substantially in biomechanical and energetic demands, and the running speeds were not metabolically matched across gradients. Therefore, combining all slope conditions in one analysis would have required a different research question and statistical approach, with slope as an additional factor, and would have made the interpretation of shoe-related effects less focused.

The present analysis includes only the uphill-running data and does not duplicate the level or downhill results. The previously published studies are cited explicitly to ensure transparency. We revised the Methods section to clarify the rationale for separate reporting and to make clear that the current study is a task-specific analysis of uphill running.

**Changes in the manuscript:**

We revised the Study design section as follows: “Following familiarization, participants completed trials under three slope conditions: 0% (level), −10% (downhill), and +10% (uphill). These conditions were collected within a broader experimental project; however, the present analysis focuses exclusively on uphill running. This decision was made because level, downhill, and uphill running represent distinct motor tasks with specific biomechanical and energetic characteristics [32–36,38,49]. In addition, running speeds were not metabolically matched across gradients [39], which limits direct interpretation of slope comparisons within a single combined analysis. Therefore, the present study was designed as a task-specific analysis of uphill running, allowing focused interpretation of shoe-related effects under this condition. Results for level [23,50] and downhill [14] conditions have been reported in previous studies.” (L 162-71)

***Reviewer’s response:***

*Line 146: Why were the speeds of 6.5km/h and 10km/h selected? 6.5km/h seems rather slow even for an uphill running assessment. You’ve cited the methods for Khassetarash et al., 2020 as a justification for these testing speeds however that study was conducted at 3 speeds with the slowest one being 9km/h at 10% incline. The speed of 6.5km/h seems too slow for meaningful effects. Please clarify reasoning for selecting these speeds.*

**Our response:**

Thank you for this comment. We agree that the rationale for selecting the speeds was not sufficiently clear. The cited graded-running study was used primarily to support the choice of a 10% incline, rather than the exact running speeds. The speeds of 6.5 and 10 km/h were selected based on pilot testing. Because running at a 10% incline substantially increased perceived exertion during repeated trials, the faster speed of 10 km/h was selected as the highest speed that was feasible for repeated uphill trials, while 6.5 km/h was selected to provide a clearly distinct lower-intensity uphill-running condition. This preserved a comparable relative separation between the slow and fast speeds used in our broader experimental protocol.

**Changes in the manuscript:**
We revised the relevant part of the Study design section as follows: “Uphill trials were performed at 6.5 and 10 km/h on a 10% incline. The 10% incline was selected based on previous graded-running literature [49]. The two speeds were selected based on pilot testing. Because running at a 10% incline substantially increased perceived exertion during repeated trials, 10 km/h was selected as a feasible higher-intensity uphill-running condition, while 6.5 km/h was chosen as a clearly distinct lower-intensity condition that allowed participants to complete all trials without fatigue.” (L 172-8)

***Reviewer’s response:***

*Line 383-385: You state the results for stability cannot be explained by sole thickness alone but rather that interactions between other shoe properties such as midsole compliance and longitudinal bending stiffness may have influenced trunk stability results. This is inconsistent with your description of the AFTs described in the methods where it is stated that “apart from sole thickness, the AFT models were identical in construction.” The thickness of the midsole may influence the overall stiffness of the shoe but this point should be clarified.*

**Our response:**

Thank you for this comment. We agree that the original wording could be misinterpreted. Our intention was not to suggest that AFT35 and AFT50 differed in midsole composition or longitudinal bending stiffness. As stated in the Methods, the two AFT shoes were identical in construction apart from sole thickness. Therefore, the absence of a significant difference in trunk stability between AFT35 and AFT50 does not support a clear thickness-dependent effect within the AFT design.

The significant trunk stability difference was observed between AFT50 and CON27. This comparison includes not only a difference in sole thickness but also the broader difference between an AFT shoe and a conventional lower-stack control shoe. We revised the Discussion to clarify this interpretation and removed the potentially confusing statement suggesting that other shoe properties explained the finding.

**Changes in the manuscript:**

**We revised the relevant sentences in the Discussion section as follows: “The reduction in trunk stability observed with AFT50 compared with CON27 indicates that the thickest AFT shoe may influence trunk stability during uphill running. However, because trunk stability did not differ between the two AFT shoes, these findings do not support the second hypothesis that greater sole thickness within the AFT design would induce larger reductions in stability. This pattern suggests that the observed difference between AFT50 and CON27 cannot be explained by sole thickness alone. Instead, it may reflect the broader comparison between an AFT shoe and a conventional lower-stack control shoe, including differences in midsole construction and longitudinal bending stiffness [5,6].” (L 414-22)**

*Reviewer #2:* ***Comments to the Author***

***Reviewer’s response:***

*ScreenIT is an independent group of scientists developing automated tools that analyze academic papers. A set of automated tools screened your submitted manuscript and provided the report below. Each tool was created by your academic colleagues with the goal of helping authors. The tools look for factors that are important for transparency, rigor and reproducibility, and we hope that the report might help you to improve reporting in your manuscript. Within the report you will find links to more information about the items that the tools check. These links include helpful papers, websites, or videos that explain why the item is important. While our screening tools aim to improve and maintain quality standards they may, on occasion, miss nuances specific to your study type or flag something incorrectly. Each tool has limitations that are described on the ScreenIT website. The tools screen the main file for the paper; they are not able to screen supplements stored in separate files. Please note that the Academic Editor had access to these comments while making a decision on your manuscript. The Academic Editor may ask that issues flagged in this report be addressed.*

**Our response:**
The authors thank the editor and reviewers for the critical and thorough revision of our work. We revised our manuscript carefully to address the criticized points. We hope that we successfully addressed all the aspects. We highlighted the new changes we made addressing the Reviewer #2’s comments with green. Please see below for our responses to each point. Please note that the line numbers are abbreviated with L and refer to the revised manuscript with tracked changes.

***Reviewer’s response:***

*Flow charts and attrition. We did not find a study flow chart of excluded observations. We strongly recommend using flow charts because they provide an overview of the study design and more information about attrition. If you included a study flow chart in your supplemental files, we apologize for missing this. Our tool is not able to screen separate supplemental files. Sentence about attrition: not detected. Please provide information about the drop-out of subjects, or loss of animals or samples. This could be done using a study flow chart, or described in the text.*

**Our response:**
Thank you for this comment. No participant drop-out occurred in the present study. All 17 recruited participants completed the uphill-running protocol and were included in the analysis. Therefore, we did not add a study flow chart, as there was no attrition to visualize. To improve transparency, we added a sentence to the Methods section explicitly stating that no participant drop-out occurred.

**Changes in the Manuscript:**

We added the following sentence in the Methods section: “All participants completed the full experimental protocol, and no participant drop-out occurred.” (L 120-1)

***Reviewer’s response:***

*Randomization. Not detected. If you performed an experimental study, please specify whether participants, animals or samples were randomly assigned to treatments or groups, and specify the randomization procedure.*

**Our response:**

Thank you for this comment. We agree that the order-assignment procedure should be stated more clearly. Because this was a repeated-measures study, participants were not randomly assigned to separate treatment groups; instead, each participant completed all shoe conditions. To minimize potential order effects, the order of the three shoe conditions was counterbalanced across participants. We have revised the Study design section to clarify this procedure.

**Changes in the Manuscript:**

We revised the relevant sentences as follows: “They then performed three counterbalanced measurement blocks, each corresponding to one standardized shoe condition. Because all participants completed all shoe conditions in this repeated-measures design, no group randomization was performed.” (L 154-7)

***Reviewer’s response:***

*Blinding. Not detected. Please specify whether blinding was used at various phases of the experiment (e.g., blinding of patients, caregivers, outcome assessments, data analysis).*

**Our response:**

Thank you for this comment. No blinding was performed in the present study. Blinding of participants and investigators was not feasible because the shoe conditions differed visibly in design and sole thickness. This is difficult to avoid in footwear intervention studies, particularly when sole thickness is a central experimental factor. We have added a statement to the Study design section to clarify this point.

**Changes in the Manuscript:**

We added the following sentence in the Methods section: “No blinding was performed, because the shoes were visibly different in design and sole thickness.” (L 157-8)

***Reviewer’s response:***

*Software and Algorithms. MATLAB suggested: MATLAB, RRID:SCR_001622. Please make sure this RRID suggestion is accurate before including in your manuscript. SPSS suggested: SPSS, RRID:SCR_002865. Please make sure this RRID suggestion is accurate before including in your manuscript. SPM suggested: SPM (RRID:SCR_007037. Please make sure this RRID suggestion is accurate before including in your manuscript.*

**Our response:**
Thank you for this suggestion. We checked the suggested RRIDs and added them to the Methods section for MATLAB, SPSS, and SPM/spm1d.

**Changes in the Manuscript:**

We revised the Data acquisition and preprocessing section as follows: “Marker trajectories were reconstructed using Vicon Nexus software (v2.15) and subsequently exported for further processing in MATLAB (R2024a; MathWorks Inc., Natick, MA, USA; RRID:SCR_001622).” (L 188)

We revised the Statistics section as follows: “Statistical analyses were performed using SPSS (Version 29.0; IBM Corp., Armonk, NY, USA; RRID:SCR_002865).” (L 228) and: “Joint angle time series were analyzed using statistical parametric mapping (SPM; RRID:SCR_007037; spm1d toolbox) implemented in MATLAB.” (L 242)

***Reviewer’s response:***

*Open code.* *If you wrote code to analyze your data, please consider sharing the code in a public repository. This makes it easier for others to reproduce your analyses, and may also aid others seeking to analyze similar datasets.*

**Our response:**

Thank you for this suggestion. We agree that sharing analysis code can improve reproducibility. However, we did not deposit the code in a public repository because the current analysis scripts are part of a broader project-specific processing pipeline and are not limited to the analyses presented in this manuscript. The Methods section describes the relevant preprocessing, event detection, nonlinear analysis, and statistical procedures in detail. To support transparency, we added a statement that the custom analysis code can be made available by the corresponding author upon reasonable request.

**Changes in the Manuscript:**

We added the following statement to the Data Availability section: “Code relevant to the analyses presented in this manuscript is available from the corresponding author upon reasonable request.” (Online submission system)

***Reviewer’s response:***

*Open data. If permitted, sharing data on a public repository with a persistent identifier (a DOI or accession number) can improve reproducibility and make it easier for other scientists to expand on your work. Papers with open data are cited more often than papers without open data. Some institutions have an expert who can provide advice on data sharing.*

**Our response:**
Thank you for this suggestion. We agree that open data supports transparency and reproducibility. The anonymized minimal dataset underlying the findings of the present study will be deposited in RADAR4KIT (<https://radar.kit.edu/radar/en/home>) upon acceptance of the manuscript. A repository DOI will be provided once available. For review purposes, the dataset is available via the following private link: https://www.radar-service.eu/radar/en/dataset/qntmuq820fay0a6p?token=vvKTjRDIhazLqDxVCnUN

**Changes in the Manuscript:**

We revised the Data Availability statement as follows: “The dataset underlying the findings of this study will be made publicly available in RADAR4KIT (<https://radar.kit.edu/radar/en/home>) upon acceptance of the manuscript.” (Online submission system)

***Reviewer’s response:***

*Selfacknowledged limitations. An explicit section about the limitations of this study was not found. We encourage authors to include a paragraph in the discussion that addresses study limitations. Every study has limitations. Describing these limitations helps readers to understand and contextualize the research.*

**Our response:**
Thank you for this comment. The manuscript already contains a dedicated **Limitations** section in the Discussion. This section addresses several study limitations, including small differences in shoe mass, treadmill-based testing, the male-only sample, uncontrolled footstrike pattern, kinematic gait-event detection, and the use of a single-segment foot model. Therefore, no additional limitations section was added. We assume that this was not detected by the automated screening tool.

**Changes in the Manuscript:**

No changes were made, as an explicit **Limitations** section was already included in the submitted manuscript. (L 438-53)
